# Supplementary material for: Spontaneous eye blink rate predicts individual differences in exploration and exploitation during reinforcement learning
Source: Sci Rep. 2019 Nov 22;9:17436. doi: 10.1038/s41598-019-53805-y (PMC6874684; doi:10.1038/s41598-019-53805-y)
Supplement: Supplementary file 1 — Supplementary Materials [file 41598_2019_53805_MOESM1_ESM.docx]

*Supplementary Information*

*Spontaneous eye blink rate predicts individual differences in exploration and exploitation during reinforcement learning*

*Joanne C. Van Slooten^1^* Sara Jahfari^2,3^ Jan Theeuwes^1^*

*^1^ Department of Experimental and Applied Psychology, Vrije Universtiteit Amsterdam, Amsterdam, The Netherlands.*

*^2^ Spinoza Centre for Neuroimaging, Royal Academy of Sciences, Amsterdam, The Netherlands.*

*^3^ Department of Psychology, University of Amsterdam, Amsterdam, The Netherlands*


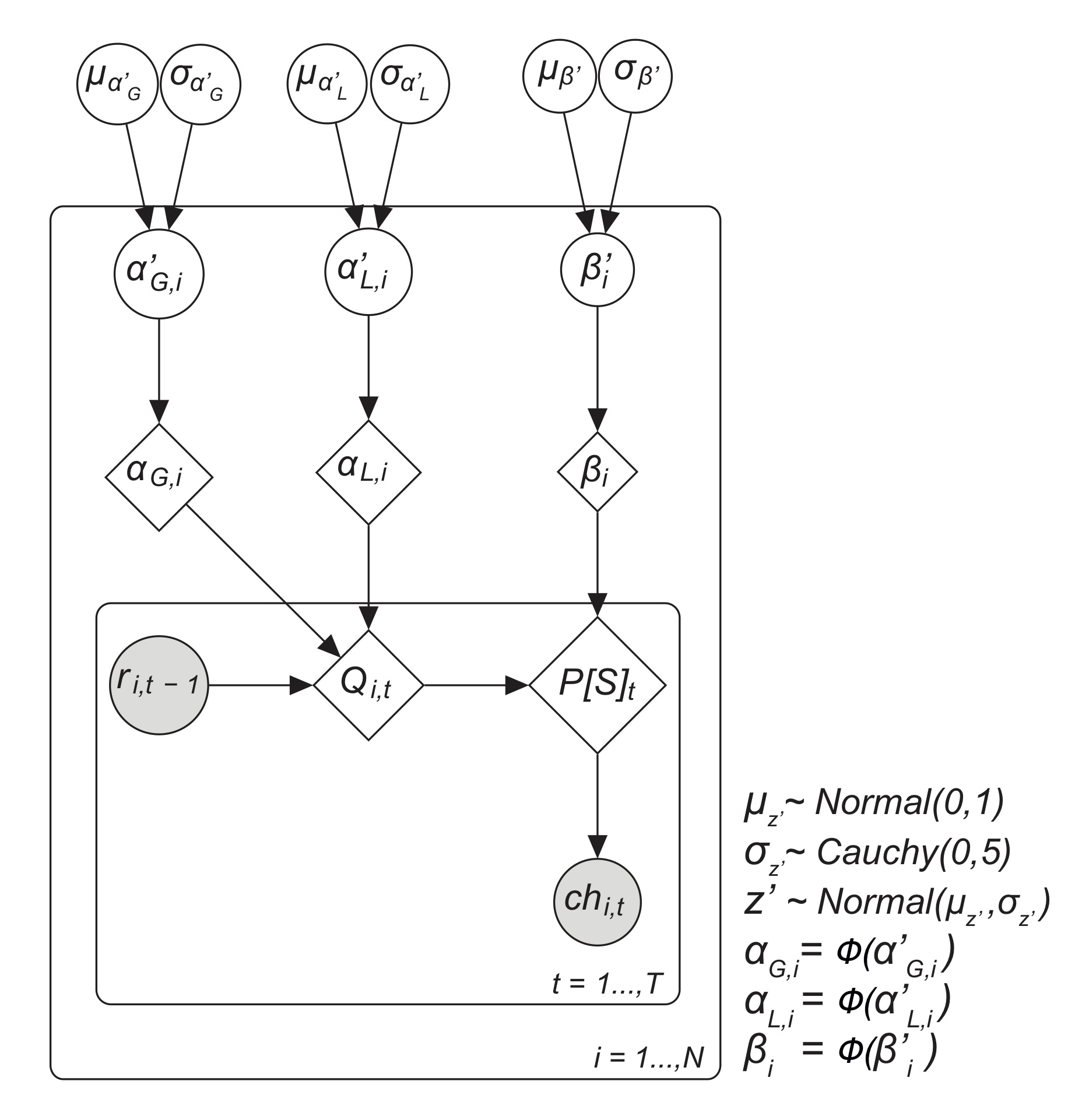


***Supplementary Figure 1.*** **Graphical representation of the hierarchical Bayesian Q-learning model.** The inner plane represents within-subject trial-by-trial RL behaviour. Variables *r_i_ (t-1)* (outcome for participant *i* on trial *t-1*) and *ch_i_(t)* (choice of participant *i* on trial *t*) were obtained from the behavioural data. The outer plane represents per-participant parameter estimates $\alpha_{\mathrm{Gi}}$ ($\alpha_{Gain}$ participant *i*),$\alpha_{Li}$ ($\alpha_{Loss}$ participant *i*) and $\beta_{i}$ ($\beta$ participant *i*) that were fit separately for participants in the low and high sEBR group. Per-participant parameter estimates were modelled using a probit transform *z’_i_* (${\alpha'}_{Gi},{\alpha'}_{Li}$, ${\beta'}_{i}$). *z’_i_* were drawn from group-level normal distributions with mean ${}_{z'}$ and standard deviation ${}_{z'}$. The outermost layer represents group-level mean and standard deviations of the Q-learning model parameters. A normal prior was assigned to all group-level means, ${}_{z'}$*~Ν(0,1),* and a half-Cauchy prior to all group-level standard deviations, ${}_{z'}$~*Cauchy(0,5).* A weakly informative prior such as this is recommended in small sample sizes to reduce the influence of the priors on posterior distributions^77^. Shaded variables are obtained from the behavioural data and used to fit the model. Diamond shaped nodes are deterministic, as they are derived from the model fit. Circular unshaded nodes indicate continuous variables. Arrows indicate dependencies between variables. *Φ()* represents the probit transform.

***
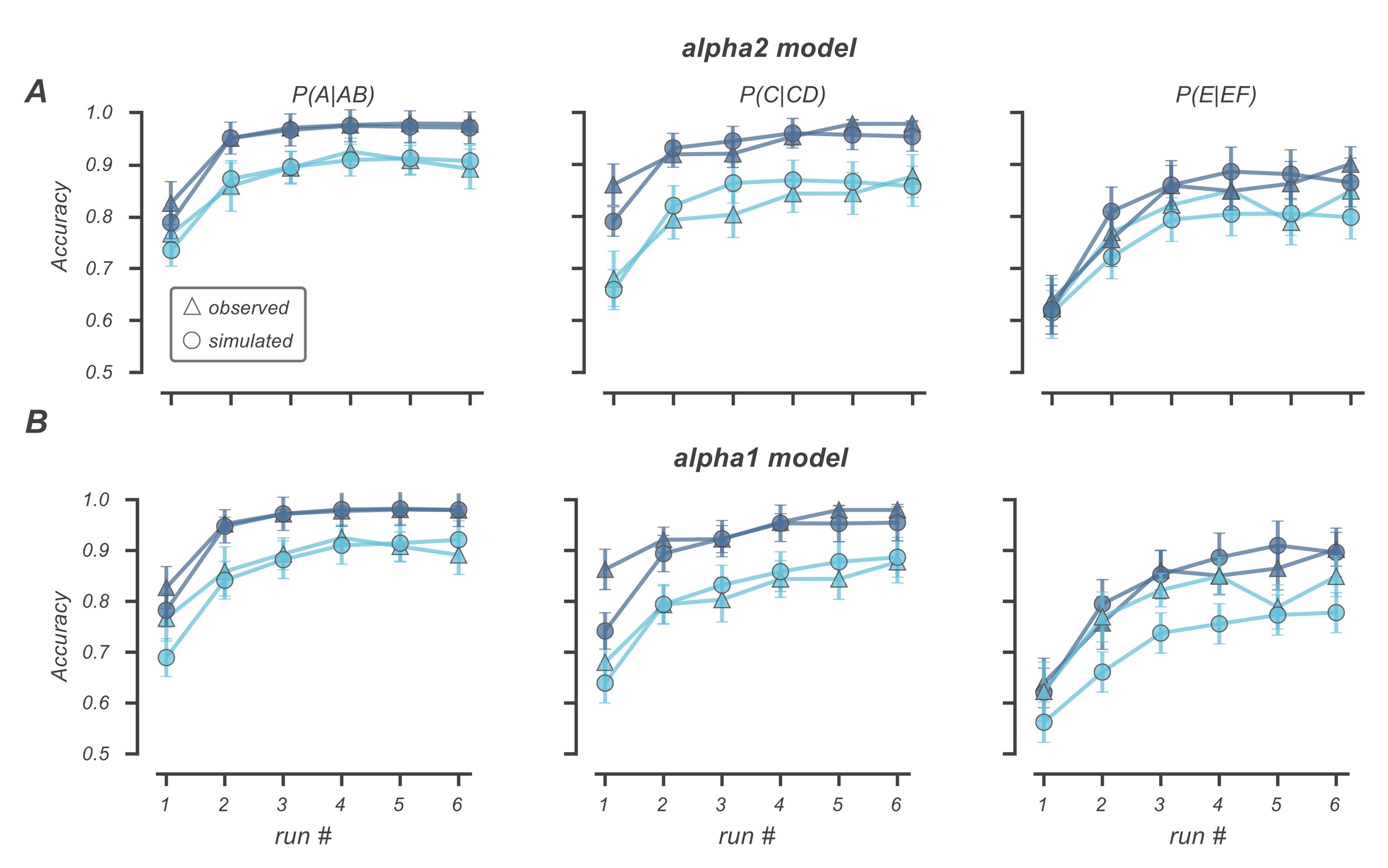
***

***Supplementary Figure 2. Posterior predictive checks (PPC) of choice accuracy learning curves for the alpha2 and alpha1 Q-learning model.*** Participants’ choice accuracy averaged across six bins with each 60 trials (observed; *triangle markers*) and plotted against simulated data (simulated; *circle markers*) by using parameter draws from the posteriors of the alpha2 (A) or alpha1 (B) model. PPC were evaluated separately for the different option pairs (AB, CD and EF) to show model performance across the three levels of choice uncertainty. The alpha1 model is the simplest Q-learning model with one learning rate (α) and one explore-exploit parameter (β). The alpha2 model has a separate α for gain and loss learning. Consistently, model comparison using PSIS-LOO indicated that the alpha2 model is most optimal to describe choices in the learning phase (elpd difference = 289.23, SD=51.98). This becomes evident when comparing both models’ simulated choices for the EF pair, where the alpha1 model consistently underestimates EF choice accuracy of high sEBR individuals. Dark blue = low sEBR; light blue = high sEBR. Error bars are SEM.

| **Model Comparison** | | | | | | | | | | | |
| --- | --- | --- | --- | --- | --- | --- | --- | --- | --- | --- | --- |
| **Models** | | **P(M)** | | **P(M\|data)** | | **BF _M_** | | **BF _10_** | | **R²** | |
| Null model |  | 0.125 |  | 0.007 |  | 0.052 |  | 1.000 |  | 0.000 |  |
| β |  | 0.125 |  | 0.353 |  | 3.814 |  | 47.859 |  | 0.297 |  |
| β + $\alpha_{Gain}$ |  | 0.125 |  | 0.314 |  | 3.205 |  | 42.618 |  | 0.349 |  |
| β + $\alpha_{Loss}$ |  | 0.125 |  | 0.196 |  | 1.710 |  | 26.634 |  | 0.327 |  |
| β + $\alpha_{Gain}$ + $\alpha_{Loss}$ |  | 0.125 |  | 0.116 |  | 0.917 |  | 15.722 |  | 0.352 |  |
| $\alpha_{Loss}$ |  | 0.125 |  | 0.006 |  | 0.045 |  | 0.868 |  | 0.071 |  |
| $\alpha_{Gain}$ |  | 0.125 |  | 0.004 |  | 0.031 |  | 0.598 |  | 0.045 |  |
| $\alpha_{Gain}$ + $\alpha_{Loss}$ |  | 0.125 |  | 0.003 |  | 0.020 |  | 0.389 |  | 0.077 |  |
|  | | | | | | | | | | | |

***Supplementary Table 1. Bayesian linear regression analysis of Q-learning model parameter modes on sEBR.*** Compared to the null model, the data provide strong evidence in favour of the model in which the β-parameter explains individual variability in sEBR.

| **Model Comparison** | | | | | | | | | | | | | | | |
| --- | --- | --- | --- | --- | --- | --- | --- | --- | --- | --- | --- | --- | --- | --- | --- |
| **Models** | **P(M)** | | | **P(M\|data)** | | | | **BF _M_** | | | **BF _10_** | | | **R²** | |
| Null model |  | 0.125 |  | | 0.048 |  | 0.350 | |  | 1.000 | |  | 0.000 | |  |
| β + $\alpha_{Loss}$ |  | 0.125 |  | | 0.338 |  | 3.574 | |  | 7.108 | |  | 0.260 | |  |
| $\alpha_{Loss}$ |  | 0.125 |  | | 0.168 |  | 1.411 | |  | 3.527 | |  | 0.159 | |  |
| β + $\alpha_{Gain}$ + $\alpha_{Loss}$ |  | 0.125 |  | | 0.134 |  | 1.079 | |  | 2.809 | |  | 0.260 | |  |
| β |  | 0.125 |  | | 0.133 |  | 1.069 | |  | 2.786 | |  | 0.145 | |  |
| β + $\alpha_{Gain}$ |  | 0.125 |  | | 0.092 |  | 0.713 | |  | 1.944 | |  | 0.185 | |  |
| $\alpha_{Gain}$ + $\alpha_{Loss}$ |  | 0.125 |  | | 0.064 |  | 0.475 | |  | 1.336 | |  | 0.161 | |  |
| $\alpha_{Gain}$ |  | 0.125 |  | | 0.025 |  | 0.177 | |  | 0.518 | |  | 0.035 | |  |
|  | | | | | | | | | | | | | | | |

***Supplementary Table 2. Bayesian linear regression analysis of Q-learning model parameter modes on avoidance accuracy in the transfer phase.*** Compared to the null model, the data provide moderate evidence in favour of the model in which both the β-parameter and $\alpha_{\mathrm{Loss}}$-parameter explain individual variability in avoidance behavior in the transfer phase.

| **Weights matrix** | | | | | | | | | | | | | |
| --- | --- | --- | --- | --- | --- | --- | --- | --- | --- | --- | --- | --- | --- |
|  | | **Network** | | | | | | | | | | | |
| **Variable** | | $\boldsymbol{\alpha}_{\boldsymbol{Gain}}$ | | $\boldsymbol{\alpha}_{\boldsymbol{Loss}}$ | | **approach** | | **avoid** | | **β** | | **sEBR** | |
| $\alpha_{Gain}$ |  | 0.000 |  | 0.522* |  | -0.016 |  | -4.340e -4 |  | -0.220 |  | -0.192 |  |
| $\alpha_{Loss}$ |  | 0.522* |  | 0.000 |  | 0.026 |  | 0.302 |  | 0.055 |  | -0.053 |  |
| approach |  | -0.016 |  | 0.026 |  | 0.000 |  | -0.043 |  | 0.139 |  | 0.037 |  |
| avoid |  | -4.340e -4 |  | 0.302 |  | -0.043 |  | 0.000 |  | 0.278 |  | -0.034 |  |
| β |  | -0.220 |  | 0.055 |  | 0.139 |  | 0.278 |  | 0.000 |  | -0.515* |  |
| sEBR |  | -0.192 |  | -0.053 |  | 0.037 |  | -0.034 |  | -0.515* |  | 0.000 |  |
|  | | | | | | | | | | | | | |

***Supplementary Table 3. Partial correlation weights matrix of all network variables.*** Asterisks indicate significant partial correlations between variables in the network.

|  |
| --- |

*Supplementary references*

77. Ahn, W-Y., Haines, N. & Zhang, L. Revealing neurocomputational mechanisms of reinforcement learning and decision-making with the hBayesDM Package. *Computational Psychiatry,* ***1****, 24-57 (2017).*
